# Tubarial glands as sentinels for environmental toxin-induced salivary dysplasia in endemic nasopharyngeal carcinoma regions

**DOI:** 10.1097/MS9.0000000000004043

**Published:** 2025-10-07

**Authors:** Abdullah Imtiaz, Muhammad Talha, Mahnoor Fatima, Omar Abdullah Gill, Muneeb Saifullah

**Affiliations:** a Department of Medicine, Wah Medical College, Wah Cantt, Pakistan; b Department of Medicine, King Edward Medical University, Lahore, Pakistan; c Department of Medicine, Shaheed Ziaur Rahman Medical College, Rajshahi Medical University, Bogura, Bangladesh

*To the Editor,*

The recently characterized tubarial glands, a pair of large salivary glands in close vicinity to the torus tubarius in the nasopharynx, have come to be critical for understanding salivary gland physiology and radiation damage in the treatment of head and neck cancers. The anatomical position of these glands, therefore, renders them directly amenable to inhaled airborne environmental toxins, particularly in endemic regions of nasopharyngeal carcinoma (NPC), wherein continuous carcinogen exposure results in gradual changes in the nasopharyngeal mucosa and malignancy. Due to the mucous gland composition of tubarial glands and their drainage pathways, they may act as early loci of toxin accumulation and dysplastic transformation, and may also mimic or precede the neoplastic changes seen in areas with endemic NPC^[1,2]^. This is in line with the TITAN Guidelines on the need for transparency in artificial intelligence use in health care^[3]^.

Nasopharyngeal carcinoma remains a major public health issue in Southeast Asia and parts of North Africa, with incidence rates above 25 per 100 000 in regions of endemicity, due chiefly to Epstein-Barr virus infection, amongst other environmental carcinogens such as nitrosamines derived from preserved food intake, and occupational airborne toxins. However, toxin-induced salivary dysplasia bears an uncharacterized role of tubarial glands; the glandular epithelium is known to be susceptible to oxidative stress and genotoxic stress. Histological examination has shown that tubarial glands consist predominantly of mucous acini expressing prostate-specific membrane antigen and possess multiple ducts draining into the nasopharyngeal cavity; therefore, these glands could serve as sentinel organs susceptible to airborne toxin exposure and early dysplastic change^[1,4]^.

Presently, public health surveillance in a number of high-exposure endemic regions is poorly augmented by assessments of changes in glandular tissues; this is largely due to logistical difficulties and ignorance regarding the tubarial glands. Such inconsistencies generate barriers for early detection of pre-malignant lesions and targeted intervention. Given that chronic exposure to toxins could result in chronic inflammation and possible metaplasia of salivary glands and could therefore cause xerostomia and dysphagia, which are frequently found in radiotherapy patients, improved surveillance with tubarial gland pathology would refine the stratification of risk for NPC^[1,5]^.

As an innovative endeavor, the analysis of tubarial gland secretions for molecules binding to toxins opens up a promising domain of biomarker study for early intervention. Newly developed toxin-binding assays for environmental carcinogens and oxidative stress markers found within glandular secretions may be able to point toward molecular precursors to dysplasia. This minimally invasive sampling may act in tandem with nasopharyngeal swabs and circulating EBV DNA studies to improve early diagnostic accuracy in endemic populations. This approach must also be integrated into public health policy, along with standardized monitoring protocols for environmental toxin exposure within susceptible cohorts^[6]^.

As a result, the discovery of the tubarial glands as sentinel glands in the salivary dysplasia caused by the environmental toxins is likely to lead to the progress of the early detection and prevention in the endemic regions of NPC. The development of assays and specific research is urgent in the context of toxicological work; to provide timely interventions, the effect of the toxicology has to be evaluated, and glandular secretions have to be measured with the help of the assays. The regular surveillance of tubarial glands of individuals who are at high risk can also be used to meet these public health objectives in eliminating morbidity and mortality associated with NPC.

## Data Availability

Not applicable to this study.

## Research Registration Unique Identifying Number (UIN)

No human subjects were involved as the type of research being submitted is an editorial.
